# Assessment of dermal bioavailability: predicting the input function for topical glucocorticoids using stratum corneum sampling

**DOI:** 10.1007/s13346-021-01064-8

**Published:** 2021-10-01

**Authors:** Andrea Pensado, Anita McGrogan, K. A. Jane White, Annette L. Bunge, Richard H. Guy, M. Begoña Delgado-Charro

**Affiliations:** 1 Department of Pharmacy & Pharmacology, University of Bath, Bath, UK; 2 Department of Mathematical Sciences, University of Bath, Bath, UK; 3 Chemical and Biological Engineering, Colorado School of Mines, Golden, CO USA; 4 Present address: R&D Cluster Programs Section, Technology Development and Innovation Center, Okinawa Institute of Science and Technology, Okinawa, Japan

**Keywords:** Betamethasone valerate, Stratum corneum sampling, Topical bioavailability, Skin blanching, Topical corticosteroids

## Abstract

Predicting the dermal bioavailability of topically delivered drugs is challenging. In this work, minimally invasive stratum corneum (SC) sampling was used to quantify the delivery of betamethasone valerate (BMV) into the viable skin. Betnovate® cream (0.1% w/w BMV) was applied at three doses (2, 5, and 10 mg cm^−2^) to the ventral forearms of 12 healthy volunteers. The mass of drug in the SC was measured using a validated tape-stripping method (a) after a 4-h “uptake” period, and (b) following a 6-h “clearance” period subsequent to cream removal. Concomitantly, the skin blanching responses to the same doses were assessed with a chromameter over 22 h post-application. BMV uptake into the SC was significantly higher for the 5 mg cm^−2^ dose compared to those of 2 and 10 mg cm^−2^. In all cases, ~30% of the drug in the SC at the end of the uptake period was cleared in the subsequent 6 h. From the SC sampling data, the average drug flux into the viable epidermis and its first-order elimination rate constant from the SC were estimated as 4 ng cm^−2^ h^−1^ and 0.07 h^−1^, respectively. In contrast, skin blanching results were highly variable and insensitive to the dose of cream applied. The SC sampling method was able to detect a 50% difference between two applied doses with 80% power; detection of a 20% difference would require a larger sample size. SC sampling enabled quantitative metrics describing corticosteroid delivery to the viable epidermis to be determined.

**Graphical abstract:**

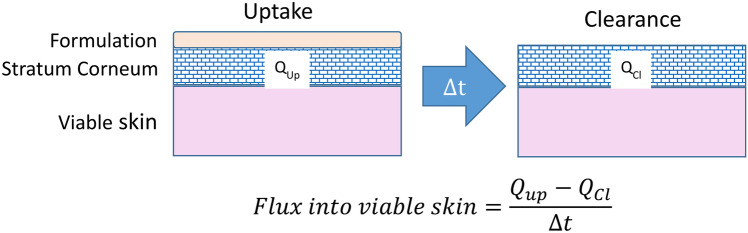

## Introduction

The pharmacodynamic response to a topically applied drug is determined by both its potency and its pharmacokinetics at the site of action in the skin [1–4]. The latter reflects the balance between the drug’s rate and extent of absorption from a given formulation to the site of action (i.e. the input function) and its clearance from the target site, for example, into the systemic circulation [5–8]. For topical corticosteroids, the input process comprises release from the applied formulation, partitioning into the stratum corneum (SC) and diffusion through the barrier to reach the viable epidermis and dermis where the target glucocorticoid receptors are located [9, 10]. Estimating the drug’s bioavailability (BA) in these tissues is complicated because its non-invasive quantification in the viable skin is challenging.

In the SC sampling approach, drug quantities in the primary barrier to drug absorption are assessed following extraction from sequentially removed tape-strips after periods of “uptake” (during application of the formulation) and “clearance” (post-removal of the drug product) [11–13]. Initial work with the method was directed at using the amounts of drug in the SC at uptake and clearance as potential metrics for the assessment of equivalence between topical formulations [12]. It was also demonstrated that the information collected could be further exploited to quantify drug input rate into the living layers of the skin (below the SC) and to provide a kinetic parameter directly relevant to topical BA [11, 13]. For example, for betamethasone valerate (BMV), it was possible to deduce, from clearance data acquired at 2, 6, and 24 h following a 6-h uptake period, that steroid elimination from the SC (and, hence, its “delivery” into the viable skin) could be described with a first-order rate constant of approximately 0.05 (± 0.02) h^−1^; in other words, about 5% of the BMV detected in the SC after a 6-h treatment was “released” into the deeper, viable epidermis per hour [14]. More recently, additional SC sampling measurements at uptake and clearance for diclofenac, acyclovir, nicotine, and lidocaine have been used not only to deduce an elimination rate constant, as for BMV, but also to estimate an average flux—in amount per unit area per unit time—into the viable skin [11, 13, 15]. At least for diclofenac and lidocaine, it appears that, while the elimination rate constant of a specific drug is independent of the product used, the flux, in contrast, is vehicle-dependent [11, 15].

The principal objective of the research presented in this paper is to examine whether the SC sampling method and data analysis approach outlined above provides an objective tool with which to quantify and permit the predictive modelling of corticosteroid BA in the skin following topical dosing. The conventional method, of course, to assess the potency and local availability of topical steroids is the vasoconstriction assay (VCA), based on measurement of the skin blanching (SB) response to these drugs [16–20]. Apart from certain biowaivers and product-specific guidances, the VCA—as described in a 1995 FDA guidance document [17]—has been for many years the only regulatory pathway to establish bioequivalence between topical drug products that did not require a clinical end-point study. The guidance stipulates a pilot dose duration—SB response study with the reference-listed drug product, to determine the appropriate dose duration, followed by a pivotal bioequivalence comparison of the “test” product with the reference [17]. Nonetheless, the VCA is subject to clear limitations that have called into question its value. First, according to some authors [2], the VCA measures drug effect on vascular tone only whereas inflammatory responses also involve redness, oedema, pruritus, cytokine release, and immune cell infiltration; indeed, an individual responding well to steroid treatment may show no skin blanching activity at all, calling into question the inferred correlation between the two [2]. Second, the FDA guidance for the VCA requires pre-screening of participants to ensure that they produce a consistent SB response that falls in the linear region of the dose–response curve [17]. Third, there is the question of dose and whether it is possible to obtain results from the VCA using clinically relevant dosing conditions for some corticosteroids [21, 22]. Finally, of course, it is evident that the VCA involves a pharmacological response and does not in any way quantify objectively the amount of drug actually transferred to the skin.

Comparative studies of SB and SC sampling have been reported and, in some cases, with good agreement (at least, up to the point where the pharmacodynamic response became saturated) [14, 23, 24]. Here, the contrast between these approaches is further investigated; SC sampling is undertaken following an optimised protocol [12]—recently recognised in a draft EMA guideline on the quality and equivalence of topical drug products [25]—using clinically relevant doses of a marketed BMV product [26–29]. Local bioavailability is quantified using metrics related to the rate and extent of absorption to the target site as described above, and the data are used to estimate the power of the technique, and the number of subjects needed, to discern between different doses. Parallel SB measurements, under the same treatment conditions, were also recorded for comparative purposes but not, it should be emphasised, with the intent of a direct head-to-head comparison with SC sampling.

## Materials and methods

### Materials

Betnovate® cream (betamethasone valerate 0.122% w/w) was purchased from Glaxo Wellcome UK Ltd (Uxbridge, UK). All doses of formulation applied in the different components of the study described below were within ± 10% of those targeted. Pure betamethasone 17-valerate, solvents, and HPLC reagents were purchased from Sigma-Aldrich (Gillingham, UK).

### Methods

#### In vivo experiments

**Fig. 1:**
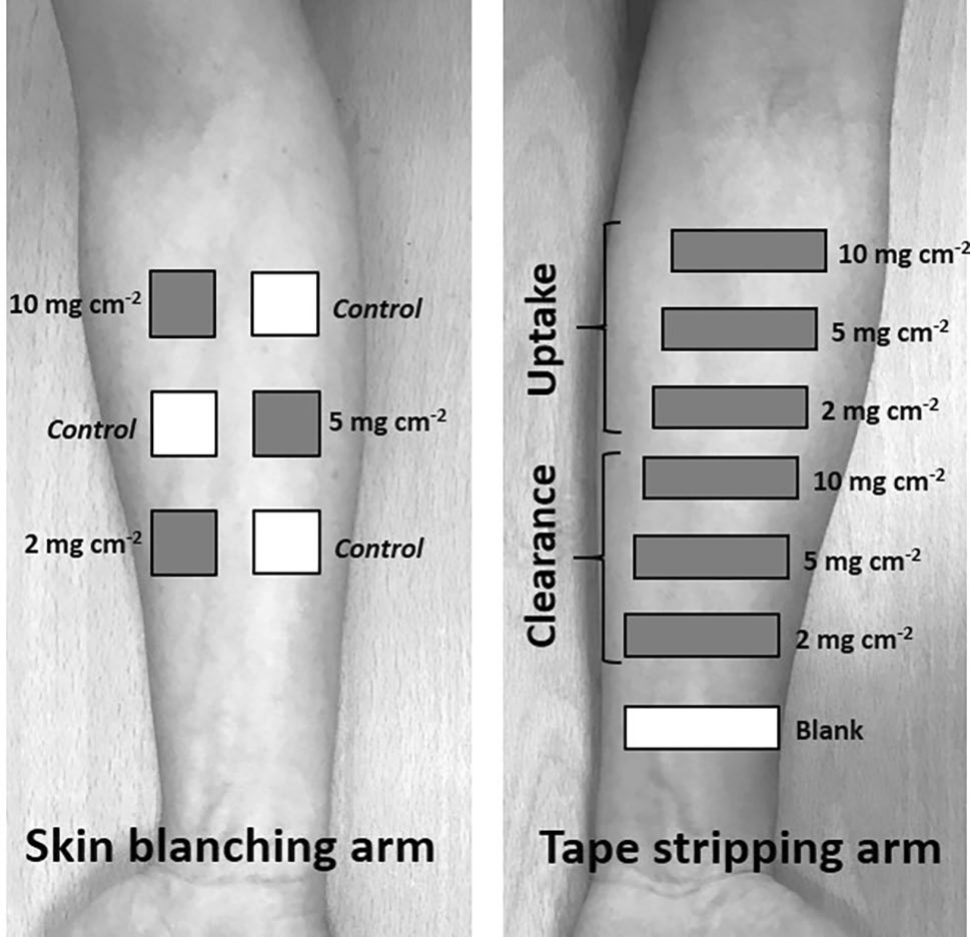
Schematic diagram of the in vivo experimental design for application of three doses of BMV cream (2, 5, and 10 mg cm^−2^) in volunteer 1. Treatment site positions in the SB assay and SC sampling experiments matched. Uptake sites in the SC sampling experiment were on the upper forearm for volunteers 1–6 and the lower forearm for volunteers 7–12. The positions of the treatment sites were rotated for each subsequent volunteer

The study was approved by the Research Ethics Approval Committee for Health at the University of Bath (REACH EP 17/18 154). Twelve healthy volunteers (7 males and 5 females), without history of dermatological disease, were enrolled and provided written informed consent. The ventral forearms of volunteers with higher hair density were shaved using a new disposable razor at least 24 h before the study began. No lotion, cream, or other personal care product was used on the volunteers’ forearms for at least 24 h prior to and throughout the study. Both arms were used in the experiments with each volunteer selecting the one for SC sampling and the one for SB (Fig. 1).

##### Skin blanching assay

The treatment sites on the SB arm (Fig. 1) were demarcated using 4 cm^2^ (2 cm × 2 cm) frames with an open area cut from self-stick adhesive (Pressure Point Foam Padding, Scholl, Slough, UK). The sites were separated at least 2.5 cm centre-to-centre and located no closer than 4 cm to the antecubital fossa or to the wrist (Fig. 1).

The higher doses of the cream were weighed into 1-mL syringes (Terumo®, Leuven, Belgium), the end section of the barrel of which was cut off at the 0.1-mL mark, and the cream was applied and spread on the skin using the plunger. The smaller doses were weighed on the top of an inverted HPLC vial which was used to spread the cream on the skin. In all cases, the mass of product applied was determined by weight difference. The doses of cream applied at the different sites were rotated from one volunteer to the next to mitigate against any potential position-dependent effects [18, 20, 30–32]. Immediately post-application, the treated sites were covered with an occlusive PVC film held on the skin by Mefix® tape (Molnlycke, Lancashire, UK). At the end of the 4-h “uptake” period, the occlusive film and the frame were removed, and the residual drug was cleaned from all the treated skin sites, first with one dry wipe (Wypall, Kimberly Clark, Kent, UK) and then with one 70% isopropyl alcohol wipe (Sterets®, Molnlycke, Lancashire, UK). Occlusion was used to optimise the otherwise poor vasoconstriction response observed in preliminary tests [18, 30, 33, 34].

Skin blanching was measured with a chromameter (CM-2600d, Konica Minolta, Warrington, UK) using the reflectance colorimeter a* scale [1, 14, 30, 32]. The “staggered application with synchronised removal” approach, described in the FDA Guidance “*Topical Dermatologic Corticosteroids – *In vivo* Bioequivalence*” [17], was used. Baseline values at the treatment and control sites (a*_T,0_ and a*_C,0_, respectively) were first acquired in duplicate before application of the BMV formulation and the pairs of results were averaged. Subsequently, after the 4-h treatment at the test sites, duplicate measurements were again made and averaged at the treated and control sites (a*_T,t_ and a*_C,t_, respectively) at 2, 4, 6, 20, and 22 h post-removal of the cream. The pharmacodynamic response profile was then characterised by the normalised change in the a* scale (Δa*_t_) defined as:

$$\Delta {a^*}_{\mathrm{t}}=\Delta {a^*}_{\mathrm{T}.\mathrm{t}}-\Delta {a^*}_{\mathrm{C},\mathrm{t}}$$

where

$$\Delta {a^*}_{\mathrm{T},\mathrm{t}}={a^*}_{\mathrm{T},\mathrm{t}}-{a^*}_{\mathrm{T},0}$$

$$\Delta {a^*}_{\mathrm{C},\mathrm{t}}=\left(\Delta {a^*}_{\mathrm{C}1,\mathrm{t}}+\Delta {a^*}_{\mathrm{C}2,\mathrm{t}}+\Delta {a^*}_{\mathrm{C}3,\mathrm{t}}\right)/3$$

and

$$\Delta {a^*}_{\mathrm{Cn},\mathrm{t}}={a^*}_{\mathrm{Cn},\mathrm{t}}-{a^*}_{\mathrm{Cn},0}$$

in which the subscript *n* designates control sites 1, 2, or 3. This analysis permits any non-drug-related impact on skin colour during the experiment to be taken into account [30]. The response (Δa*_t_), plotted as a function of time for each volunteer and for each dose, logically decreased with increasing skin blanching. The data are therefore presented as the “area above the blanching effect curve” (AAEC) from 0 to 22 h (i.e. area between the *x*-axis and the experimental curve) which was calculated using the trapezoidal rule.

##### In vivo SC sampling

Betnovate® cream was applied in duplicate at 2, 5, and 10 mg cm^−2^ to 6 treatment sites on the SC sampling arm (Fig. 1), and the mass of drug in the SC was measured at one “uptake” (4 h) and at one “clearance” (6 h) time point. The treatment sites were demarcated using rectangular-shaped frames with an 8.25 cm^2^ (1.5 cm × 5.5 cm) open area cut from self-stick adhesive (Pressure Point Foam Padding, Scholl, Slough, UK). The sites were separated by 1.6 cm and located at least 5 cm above the wrist and a minimum of 0.5 cm below the antecubital fossa. Positions of the three application sites matched those of the SB assay. Uptake sites were assigned to the upper forearm in volunteers 1–6 and to the lower forearm in volunteers 7–12.

Higher and lower doses of the cream were weighed and applied to the skin as described for the SB assay. Immediately post-application, the treated sites were covered with an occlusive PVC film held on the skin by Mefix® tape (Molnlycke, Lancashire, UK). At the end of the 4-h “uptake” period, the occlusive film and the frame were removed, and the residual drug was cleaned from all treated skin sites as described above.

SC was sampled at all designated uptake sites immediately after cream removal. The edges of the treatment areas on the clearance section were demarcated using Mefix® tape without encroaching on the treated area. The clearance sites on the forearm were covered with light gauze (Boots, Nottingham, UK) to protect the area for 6 h until SC sampling was performed. Immediately prior to SC sampling, a thin template of Scotch® Book Tape (3 M, St. Paul, MN, USA) was used to define a central 5 cm^2^ area (1 cm × 5 cm) of the drug application sites. These sites were then tape-stripped by repeated application of Book Tape (1.5 × 6.5 cm) that overlapped the edges of the template. Each tape was pressed firmly to the skin, with rubbing for a few seconds, and then removed in alternating directions for successive strips. Transepidermal water loss (TEWL) was measured (Model AF200 AquaFlux® evaporimeter, Biox System Ltd., London, UK) before and during the tape-stripping process to identify when most of the SC was removed without causing too much discomfort to volunteers. Tape-stripping was stopped if any one of the following occurred: (a) TEWL reached 60 g m^−2^ h^−1^, (b) the TEWL value exceeded 6 times the baseline pre-stripping value, or (c) 20 tapes had been removed. A maximum of 20 tapes was used in this study because preliminary results showed that, beyond this point, either no drug was found, or the amount present was below the limit of quantification. Twenty tape strips were collected from all (36) clearance sites and from all but 6 uptake sites. The mass of SC removed was determined by weighing the tapes (Microbalance SE-2F, precision 0.1 µg; Sartorius AG, Göttingen, Germany) before and after tape-stripping; to facilitate accurate measurements, tapes were discharged of static electricity (R50 discharging bar and ES50 power supply Eltex Elektrostatik GmbH, Germany) before being weighed.

BMV was extracted from groups of tape-strips into 2 mL of 60:40 v/v acetonitrile (ACN):water by sonication for 1 h followed by shaking overnight at room temperature. Samples were filtered (0.45-µm nylon membrane, SMI-Labhut, Ltd., Maisemore, UK) and transferred to HPLC vials for analysis. The tape-strips were grouped to ensure that the aggregated samples contained a sufficient drug amount to exceed the limit of quantification of the assay. Typically, the first two tape-strips were extracted together, while the remainder were combined into 3 groups of 6 tapes. Control samples of SC (indicated by “blank” in Fig. 1) that had not been exposed to any BMV-containing formulation were acquired from each volunteer and subjected to the identical extraction and analysis procedures to confirm the absence of any interference in the chromatogram at the retention time of the drug.

### HPLC analysis

BMV extracted from SC on the tape strips was quantified by HPLC (Shimadzu LC-2010, Buckinghamshire, UK) with UV detection (240 nm). A mobile phase of 60:40 ACN:water was pumped at a flow rate of 1 mL min^−1^ through a 250 × 4.6 mm HiQ Sil C18 column (Kromatek, Dunmow, UK) at 25 °C. The injection volume was 50 µL and the retention time of BMV was ~ 11.6 min; limits of quantification and detection were 0.032 and 0.01 µg mL^−1^, respectively. LC–MS (Bruker Daltonik GmbH, Bremen, Germany) confirmed the identity of the BMV peak and the absence of BMV metabolites in the samples.

### Data analysis

The thickness of the SC removed by tape-stripping was calculated from the mass of SC on each tape divided by the area sampled and assuming the density of the SC is 1 g cm^−3^ [35]. Average concentration of drug in the SC of the extracted groups of tapes was the ratio of the extracted drug divided by the SC mass on these tape(s). The average flux of drug transferred from the SC to the underlying tissue (*J*) during “clearance” for each applied dose was calculated from:

1 $$J=\left({Q}_{\mathrm{Up}}-{Q}_{\mathrm{Cl}}\right)/\Delta t$$

where *Q*_Up_ is the mass per unit area of drug in the SC at the end of the 4-h “uptake”, *Q*_Cl_ is the mass per unit area of drug in the SC 6 h after removal of the residual formulation, and Δ*t* is the elapsed time between the “uptake” and “clearance” measurements, i.e. 6 h. Assuming that BMV is cleared from the SC with first-order kinetics, then the associated elimination rate constant (*k*) is defined as:

2 $$k=-\mathrm{ln}\left({Q}_{\mathrm{Cl}}/{Q}_{\mathrm{Up}}\right)/\Delta t$$

Grubbs’ test was performed to detect significant outlier values using GraphPad. Statistically significant differences were estimated (as appropriate) by either a two-tail *t*-test or a one-way ANOVA or a repeated measures ANOVA, followed by Tukey’s test. In all the comparisons undertaken, statistical significance was set at *p* < 0.05.

### Power analysis and sample size calculation for SC sampling and the SB assay

To inform the interpretation of this study and for future work, sample size calculations and power analyses were undertaken. Power was set to be 80% and the required sample sizes were determined for doses of 2, 5, and 10 mg cm^−2^. A power analysis was also conducted to determine whether the design of the current investigation was able to detect a 20% or 50% change in mean responses of *Q*_Up_, *Q*_Cl_, and AAEC 0–22 h. In this way, the aim was to estimate the number of volunteers necessary to detect a 20% or 50% change in the three parameters assumed to be metrics of dermal bioavailability. For AAEC 0–22 h, the analysis was performed twice: first, using data from volunteers showing a measurable response (AAEC 0–22 h < 0) at any of the Betnovate® doses—that is, independent of whether the volunteer did or did not respond to other doses—second, using results only from the volunteers who responded to all three doses and could therefore be considered as “more consistent” responders. All analyses were conducted using Stata version 15 with alpha set to 0.05.

## Results

### In vivo SC sampling

**Fig. 2:**
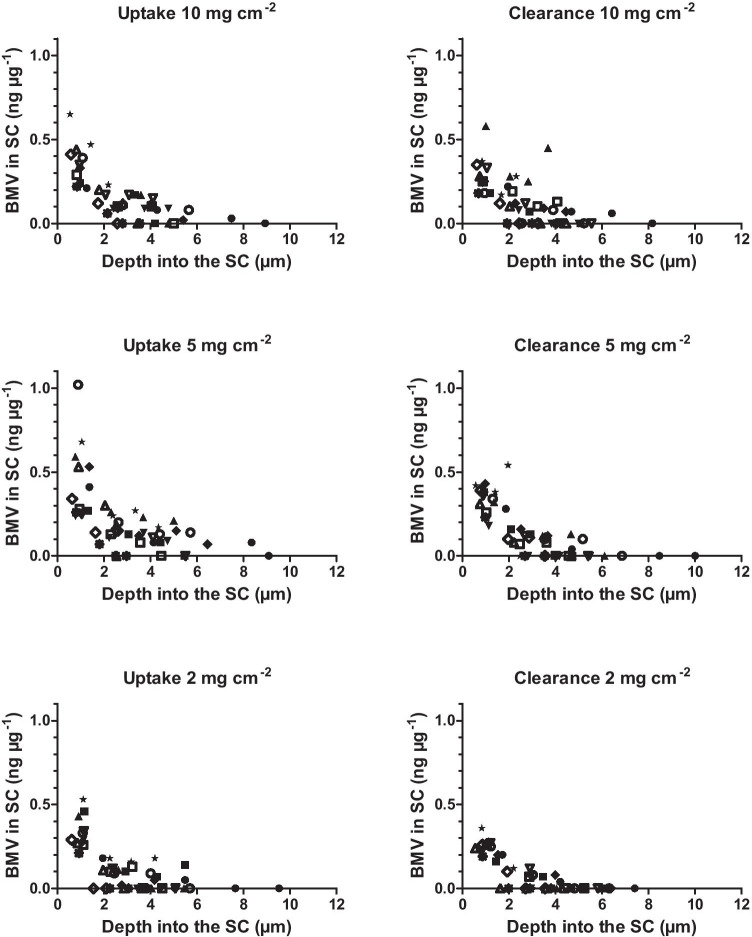
BMV concentration profiles versus depth in the SC for each of the 12 volunteers (designated by a different symbol) after a 4-h uptake period and following 6 h of clearance post-removal of the formulation applied at 2, 5, and 10 mg cm^−2^. Note that the concentrations shown are, in fact, the average values in the corresponding SC mass of each group of tape strips and are plotted against the cumulative depth into the SC

Twelve healthy volunteers participated and completed the study. There were no significant differences between the masses of SC sampled from the treated skin sites either for the different doses applied or for those used for uptake and clearance measurements. The average SC thickness removed was 4.9 ± 1.6 µm (*n* = 72) which corresponds to 0.49 mg of SC per cm^2^. The disposition of BMV in the SC at the uptake and clearance times, following application of the three doses of the cream, is shown in Fig. 2.

**Fig. 3:**
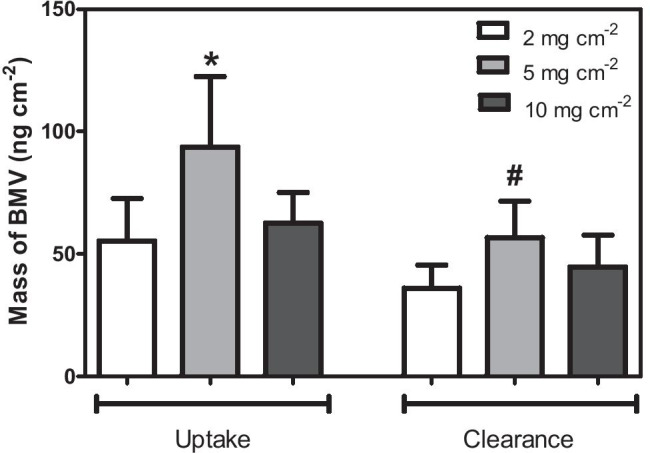
Mass of BMV in the SC at uptake and clearance following application of three different doses of the Betnovate® formulation. Data show the mean (+95% confidence interval (CI)) for *n* = 12 (except at 10 mg cm^−2^ clearance for which *n* = 11 due to a significant outlier). *The 5 mg cm^−2^ value is significantly greater (*p* < 0.05) than those for 2 and 10 mg cm^−2^. ^#^The 5 mg cm^−2^ value is significantly greater (*p* < 0.05) than that for 2 mg cm^−2^

The total masses of BMV in the SC at uptake and clearance (*Q*_Up_ and *Q*_Cl_, respectively) post-treatment with the three different doses of Betnovate® are presented in Table 1 and Fig. 3. The BMV amount at uptake after the 5 mg cm^−2^ dose was statistically significantly higher (*p* < 0.05) than those following treatment at 2 and 10 mg cm^−2^. At clearance, the mass of BMV at the 5 mg cm^−2^ site was significantly greater than that at the 2 mg cm^−2^ site, but was not different from that at the 10 mg cm^−2^ site. For all doses, the BMV quantity in the SC was higher (*p* < 0.05) at uptake than that at clearance; the average relative depletion of BMV from the SC during clearance was 33%, 37%, and 28% for the 2, 5, and 10 mg cm^−2^ doses, respectively. Table 1 also reports the deduced average flux of BMV into the underlying viable tissue during the clearance period (*J* determined from Eq. 1), and the first-order rate constant (*k* estimated from Eq. 2) describing BMV clearance from the SC. There were no statistically significant differences between the values of either *J* or *k* at the different doses of formulation.

**Table 1:** SC sampling results. BMV measured in the SC following uptake and clearance periods (*Q*_Up_ and *Q*_Cl_, respectively), the deduced drug flux into the underlying viable tissue (*J* determined using Eq. 1), and the associated elimination rate constant (*k* calculated from Eq. 2). Data are the arithmetic means ± 95% CI from 12 volunteers (except for *Q*_Cl_ at a dose of 10 mg cm^−2^ for which *n* = 11 due to one significant outlier)

**Dose of formulation (mg cm**^−**2**^**)**	**2**	**5**	**10**
*Q*_Up_ (ng cm^−2^)	55 ± 17	94 ± 29	63 ± 12
*Q*_Cl_ (ng cm^−2^)	37 ± 9	59 ± 14	45 ± 13
*J* (ng cm^−2^ h^−1^)	3 ± 3	6 ± 3	3 ± 2
10^2^ k (h^−1^)	6 ± 5	7 ± 3	6 ± 4

### Skin blanching assay

**Fig. 4:**
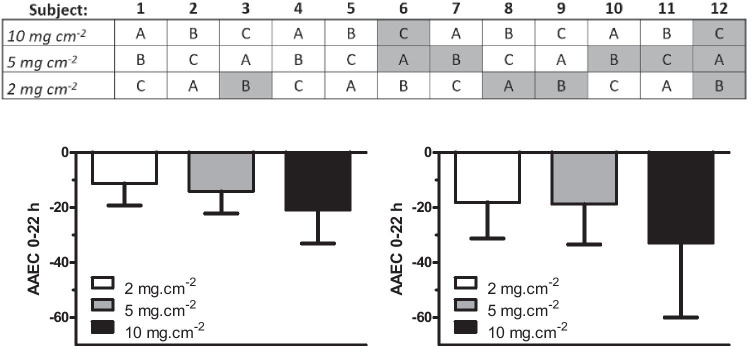
The upper panel shows the rotated positions of the different doses in the 12 human volunteers to different sites on the ventral forearm: A, closer to the elbow; B, middle; C, closer to the wrist; shading of the individual cells in this graphic indicates either a positive (white) or no (grey) pharmacodynamic response to BMV. The bottom left panel presents the AAEC 0–22 h results when a measurable change in the a* scale was observed (mean, −95% CI, *n* = 7–10); the bottom right panel reports the corresponding data from only those 4 volunteers who responded to all doses of the formulation (mean, −95% CI, *n* = 4) [note that AAEC has units of “hours”]

The pharmacodynamic response to BMV following a 4-h application of the three doses of Betnovate® was measured in 12 volunteers over a period of 22 h post-removal of the formulation. The vasoconstriction observed was highly variable, an unsurprising outcome given that the volunteers had not been pre-screened to confirm their ability to meet the criteria for inclusion in a formal vasoconstriction assay as defined by the regulatory guidance [17, 18, 30, 34]. In fact, all volunteers bar one responded to at least one of the doses applied with 8 volunteers reacting to the 2 mg cm^−2^ dose, 7 to the 5 mg cm^−2^, and 10 to the 10 mg cm^−2^. However, only 4 volunteers showed skin blanching to the three doses. Figure 4 summarises this information and presents the SB data in terms of the derived AAEC 0–22 h values. The results are presented in two ways: first, the left panel includes all the results when volunteers showed a measurable response (even if this was to only one or two of the doses administered); second, the right panel includes the data from just those 4 volunteers who responded to every dose. Regardless of the approach followed, no statistically significant differences were found in the AAEC 0–22 h responses to the three different doses of the cream.

### Power analysis and sample size estimation

The results of these analyses are summarised in Table 2 (for power (1 − β) and a significance level *α* set to 0.05 for the sample size calculations). The SC sampling experiments were powered sufficiently to detect a 50% change in the mean response for uptake (*Q*_Up_) and clearance (*Q*_Cl_) at all doses ((1 − β) range: 88.7–99.9%), but not to detect a 20% change in response in these metrics ((1 − β) range 24.7–52.1%). In contrast, the SB assay was not powered enough to detect either a 20% ((1 − β) < 12% in all cases) or a 50% ((1 − β) < 44% in all cases) change in the mean AAEC 0–22 h in the two population subsets considered, i.e. “responders to one or more dose” and “responders to all three doses”.

**Table 2:** Power analysis and sample size calculations for both SC sampling uptake (*Q*_Up_) and clearance (*Q*_Cl_) and SB (AAEC 0–22 h) metrics. For the SB response, the analyses were done for two population subsets: “responders to one or more dose” (*n* = 8 for 2 mg cm^−2^, *n* = 7 for 5 mg cm^−2^, and *n* = 10 for 10 mg cm^−2^) and “responders to all three doses” (*n* = 4). All analyses were conducted with *α* set to 0.05 and for the sample size calculation, power (1 − β) was 80%

**SC sampling**							
	**Uptake (*Q***_**Up**_**)**				**Clearance (*Q***_**Cl**_**)**		
Dose of cream (mg cm^−2^)	2	5	10		2	5	10
*n*	12	12	12		12	12	11
Mean	55	94	63		43	69	48
Standard deviation	27	45	20		20	32	17
20% change in mean	11	19	13		9	14	10
50% change in mean	28	47	31		22	34	24
	**Sample size required to detect indicated change in mean**						
20% change in mean	51	49	22		49	44	28
50% change in mean	10	10	6		10	9	7
	**Power (1 − β) of current study to detect indicated change in mean**						
20% change in mean	24.7%	25.7%	52.1%		33.7%	34.5%	51.4%
50% change in mean	88.7%	90.1%	99.9%		96.9%	97.3%	99.9%
**Skin blanching response**							
**Volunteers**	**Responders to ≥ 1 dose**				**Responders to all 3 doses**		
Dose of cream (mg cm^−2^)	2	5	10		2	5	10
*n*	8	7	10		4	4	4
Mean	−11.4	−14.2	−20.9		−18.2	−18.8	−33.0
Standard deviation	9.6	8.8	17.0		8.2	9.3	17.0
20% change in mean	−2.3	−2.8	−4.2		−3.6	−3.8	−6.6
50% change in mean	−5.7	−7.1	−10.5		−9.1	−9.4	−16.5
	**Sample size required to detect indicated change in mean**						
20% change in mean	143	77	133		43	50	55
50% change in mean	25	14	23		9	10	11
	**Power (1** − **β) of current study to detect indicated change in mean**						
20% change in mean	9.0%	11.3%	10.7%		9.8%	9.1%	8.7%
50% change in mean	30.5%	43.7%	41.1%		33.7%	29.6%	27.5%

The results obtained also permitted estimation of the sample sizes required to detect either a 20% or 50% change in the surrogate markers for BMV topical bioavailability (i.e. *Q*_Up_, *Q*_Cl_, and AAEC 0–22 h). For *Q*_Up_ and *Q*_Cl_, 6–10 volunteers would permit a 50% change to be demonstrated; to show a 20% change in the mean, however, would require 22–51 volunteers. For the SB metric, the number of volunteers needed to detect either a 20% or a 50% change in AAEC 0–22, assuming a volunteer population of individuals that responded to one or more dose of the BMV cream, was high: 77–143 and 14–25 volunteers, respectively. If the participants were pre-screened to include only those people who responded to all 3 doses of the drug product then, to detect a 20% or a 50% change, 43–55 and 9–11 volunteers would be needed, respectively. These estimations are consistent with those made for *Q*_Up_ and *Q*_Cl_; however, in the case of SC sampling, no pre-selection of volunteers is necessary.

## Discussion

The therapeutic effect of a dermatological drug (such as BMV) in a suitable formulation (e.g. Betnovate® cream) depends upon both the pharmacological potency of the active and its local pharmacokinetics in the skin. The latter, including the all-important absorption of the drug to the target site, are functions of the compound’s physicochemical properties and the vehicle in which it is applied [1, 4, 36–38]. The SB response to corticosteroids, including BMV, has been used for many years as a surrogate measurement of topical bioavailability despite the debate as to whether the assay is truly correlated with the anti-inflammatory effect of this drug class [2], the non-linear relationship between local drug concentration and SB [14, 18, 36, 39], the lack of SB response shown by a subset of the population [17, 18, 36], the (non-clinical) use of occlusion in such studies [14, 18, 39], the application of drug product doses exceeding those which are considered clinically relevant, and other concerns [32, 36, 40]. These shortcomings have been reinforced by the research presented here. In contrast, SC sampling has been shown to offer an objective and quantitative approach to assess drug uptake into and clearance from the skin’s barrier layer, at topical doses used by the patient (i.e. 2–10 mg cm^−2^) [26–29].

The study design aimed to optimise the assessment of the surrogate dermal bioavailability metrics for BMV. Thus, the SC sampling uptake time of 4 h, with clearance measured 6 h later, ensured (a) that the quantity of drug in the SC was easily quantifiable and that a significant depletion of BMV occurred during the clearance phase [12, 14], and (b) that the protocol was acceptable—in terms of convenience and time commitment—to the volunteer participants. Also, in preliminary in vitro permeation experiments, the masses of BMV in the SC after 2-, 4-, and 6-h applications were essentially the same (data not shown). With respect to the timing of the SB experiments, the longer application time of 6 h was selected to ensure that the maximum vasoconstriction response had been achieved [30, 41] even though some earlier reports have suggested that 4 h is sufficient for this purpose [14, 41]. The period, over which SB was followed post-removal of the BMV cream, was consistent with previous experience [14] and with the FDA guidance [17].

The SC sampling experiments, based on a cohort of volunteers, met the objective of unequivocal quantification of the amount of BMV in the barrier layer of the skin. The *Q*_Up_ from a 5 mg cm^−2^ dose of the cream was significantly higher (2-way ANOVA followed by Tukey’s multiple comparison test) than that following application of either the 2 or the 10 mg cm^−2^ doses; there was no significant difference between *Q*_Up_ values from the smallest and highest doses of the formulation. The smaller BMV uptake at 10 mg cm^−2^ compared to 5 mg cm^−2^ was an unexpected finding. One hypothesis, reserved for a future investigation, is that occlusion caused the composition and/or structure of the residual phase on the skin after application, from which drug delivery occurs [43], to be different for the two thicknesses. However, the percentage of the BMV applied, which was actually recovered from the SC after the 4-h uptake was relatively small: 2.76 ± 1.37, 1.87 ± 0.91, and 0.63 ± 0.20%, respectively, for the 2, 5, and 10 mg cm^−2^ doses; furthermore, the *Q*_Up_ values at different doses varied, on average, by less than a factor of two. In other words, despite the significant differences that were observed and which the SC sampling method was able to detect, the impact of the different cream doses used—in terms of drug delivery to the skin—was relatively small. In terms of *Q*_Cl_, the only significant difference identified was that the value from a 5 mg cm^−2^ dose was greater than that from the 2 mg cm^−2^ application. The differences between the average values of *Q*_Cl_ at the three cream doses was (like *Q*_Up_) modest and no more than a factor of 1.6.

Consistent with these observations are the average fluxes (*J*) of BMV from the SC into the viable epidermis, and the associated rate constants (*k*) describing drug elimination from the SC (Table 1), deduced from Eqs. 1 and 2, respectively, as a function of formulation dose. Neither the values of *J* (3 to 6 ng cm^−2^ h^−1^) nor *k* (0.06–0.07 h^−1^) were statistically significantly different between doses. The results for *k* were in good agreement with a previous investigation, which reported *k* = 0.04–0.06 h^−1^, involving infinite doses of BMV applied from two vehicles that were distinctly different from Betnovate® cream [14]. This insensitivity of *k* to the nature of the applied formulation has been reported recently for diclofenac, acyclovir, and lidocaine [11, 13, 14]. However, in these instances (and also in the earlier BMV study mentioned above [14]), the nature of the formulation did significantly impact on the measured *Q*_Up_ and *Q*_Cl_ and, hence, on the deduced fluxes (*J*). Logically, formulation excipients are expected to affect key parameters that determine the efficiency of drug delivery, not the least of which are partitioning and solubilisation of the drug in the SC and its diffusivity across the barrier. Precisely how this takes place and which factor predominates is the subject of current research; of particular focus is the transformation, or metamorphosis, of the drug product as it is rubbed into the skin [42] and the role of the residual phase that remains once volatile and/or rapidly taken-up components of the formulation have dissipated. The SC sampling results reported here and previously are contributing to unpicking the drug’s “input function” from the applied product, an essential element to creating a predictive model of dermatopharmacokinetics. Such a capability would then be combined with information on drug clearance from the skin [5–8, 13] to provide a tool with which to optimise a formulation and maximise the residence time of the active at its therapeutic target.

The SB experiments were undertaken in the same 12 volunteers. As anticipated, variability in the pharmacological response was considerable and only 4 volunteers had measurable SB to all three doses of the formulation (Fig. 4). No statistically significant differences between the responses at different doses were observed (whether data from all 12 volunteers, or from just the 4 “consistent” responders were compared). As a consequence, no direct correlation with the SC sampling results was found in terms of *Q*_Up_ and *Q*_Cl_, *J*, and *k*. Although correlations between BMV uptake into the SC and vasoconstriction response have been reported in the literature, these studies have invariably pre-selected participants, who showed consistent blanching, and involved higher doses of the drug [14, 18, 23, 24, 39]. However, even in these studies, saturation of the pharmacodynamic response was eventually observed despite the fact that SC uptake continued to increase with increasing BMV thermodynamic activity in the formulation [14, 18, 39]. Parenthetically, it has been pointed out that the white soft paraffin in Betnovate® cream [29] may itself contribute to the observed vasoconstriction, further confounding the interpretation of the SB results [40].

It has been argued that variability in the SB assay is primarily due to inter-subject differences in SC permeability to the steroid (and, hence, to differences in local bioavailability) [34]. In parallel, while agreeing that the intensity of the SB response may be a predictor of percutaneous absorption, it has been opined that vasoconstriction per se has little, if anything, to do with clinical outcome [2]. The research reported in the present paper makes a further contribution to this debate and shows that quantitative metrics on BMV uptake into and clearance from the SC, as well as an estimate of flux into the viable epidermis, can be accessed using SC sampling in all the volunteers who participated in the study. In contrast, in the SB experiments, only one-third of the cohort responded consistently to all three doses of the BMV cream, clearly illustrating exactly why the vasoconstriction assay guidance [17] requires pre-selection to be performed in a pilot study before the pivotal investigation is initiated.

It follows that the ability of SC sampling to reliably provide quantitative measures of topical bioavailability under experimental conditions directly relevant to clinical use—and potentially offering mechanistic insight into formulation performance [3]—coupled with no requirement or need to pre-select only “responding” participants, is a significant advantage over the classic vasoconstriction assay. This conclusion is reinforced by the results in Table 2, which compares the power of the SC sampling approach and the SB assay, and the sample sizes required, to detect either 50% or 20% changes in the respective metrics (i.e. *Q*_Up_ and *Q*_Cl_ versus AAEC 0–22 h). For SC sampling, the power to detect a 50% change in the means of *Q*_Up_ and *Q*_Cl_ was > 80% with only 12 volunteers; in comparison, the corresponding power of the AAEC 0–22 h metric was never more than 45%. As a result, about 3 times more participants would be needed for the SB assay to provide the same discriminatory power as a SC sampling protocol. For both SC sampling and the SB assay, detection of a 20% change in the key metrics, i.e. closer to what might be necessary to establish bioequivalence between two drug products, would require about 3–fivefold and 4–sixfold additional volunteers (relative to those needed to elicit a 50% change), respectively.

In summary, this work and previous research [11–13, 15, 28, 43] suggest that metrics derived from a well-designed SC sampling study can be used to deduce the input rate of a drug across the skin and thereby provide information pertinent to either topical or transdermal bioavailability. Assuming that clinically relevant SC concentrations of the drug of interest can be measured and that sufficient SC clearance occurs in up to a 24-h period post-removal of the formulation (so as to avoid any significant drug loss by desquamation), the technique offers a simpler, less expensive, and minimally invasive approach to screen formulations during product development, to establish the impact of critical parameters in a quality-by-design approach, and to assess post-marketing safety and efficacy in different user populations, in real-world studies, and following single and multiple dosing.

## Conclusion

This study confirms that SC sampling is an objective, reproducible, and valid method with which to measure key parameters that characterise corticosteroid bioavailability in the skin. Compared to the conventional vasoconstriction assay, SC sampling has more discriminatory power, significantly less variability, particularly under clinically relevant dosing conditions, and requires smaller sample sizes by having no need to pre-select “consistent” responders for the experimental cohort.

## Data Availability

The datasets generated during the current study are not publicly available as approval is required for secondary data analysis by third parties.

## Declarations

### Animal studies

No animal studies were carried out by the authors for this article.
